# Low levels of PCSK9 are associated with remission in patients with rheumatoid arthritis treated with anti-TNF-α: potential underlying mechanisms

**DOI:** 10.1186/s13075-020-02386-7

**Published:** 2021-01-19

**Authors:** Johan Frostegård, Sabbir Ahmed, Ingiäld Hafström, Sofia Ajeganova, Mizanur Rahman

**Affiliations:** 1 Section of Immunology and Chronic Disease, Institute of Environmental Medicine, Karolinska Institutet, Nobels väg 13, IMM, 17177 Stockholm, Sweden; 2 Division of Gastroenterology and Rheumatology, Department of Medicine Huddinge, Karolinska Institutet, Stockholm, Sweden; 3 Rheumatology Unit, Karolinska University Hospital, Stockholm, Sweden; 4 Rheumatology Division, Universitair Ziekenhuis Brussel, Vrije Universiteit Brussel, Brussels, Belgium

**Keywords:** Proprotein convertase subtilisin kexin 9 (PCSK9), Rheumatoid arthritis, Disease activity, Tumor necrosis factor (TNF), Macrophages, Synoviocytes

## Abstract

**Background:**

Proprotein convertase subtilisin kexin 9 (PCSK9) targets the LDL-receptor (LDLR) which raises LDL-levels. In addition, PCSK9 has proinflammatory immunological effects. Here, we investigate the role of PCSK9 in relation to the inflammatory activity in patients with rheumatoid arthritis (RA).

**Methods:**

PCSK9-levels were determined at baseline by ELISA in 160 patients with RA not previously treated with biologics. The patients started anti-TNF-α (adalimumab, infliximab, or etanercept) treatment and were followed-up for 1 year. Disease activity was determined by DAS28.

Effects of PCSK9 on cytokine production from macrophages of healthy individuals and synoviocytes from RA patients and inhibition by anti-PCSK9 antibodies were studied in supernatants by ELISA.

**Results:**

A significantly lower level of PCSK9 at baseline, *p* = 0.035, was observed in patients who reached remission within 1 year, defined as DAS28 < 2.6, compared to those not in remission. At 12 months of TNF-α antagonist treatment, the mean DAS28 was reduced but was significantly greater in patients with highest quartile PCSK9 (Q4) compared to those at lowest PCSK9 (Q1) in both crude (*p* = 0.01) and adjusted analysis (*p* = 0.004).

In vitro, PCSK9 induced TNF-alpha and IL-1beta in macrophages and monocyte chemoattractant protein-1 (MCP1) in synoviocytes. These effects were inhibited by anti-PCSK9 antibodies.

**Conclusions:**

Low levels of PCSK9 at baseline are associated with being DAS28-responder to anti-TNF-α treatment in RA. An underlying cause could be that PCSK9 stimulates the production of proinflammatory cytokines from macrophages and synoviocytes, effects inhibited by anti-PCSK9 antibodies. PCSK9 could thus play an immunological role in RA.

## Introduction

Rheumatoid arthritis (RA) affects 0.5 to 1% of total population, placing a substantial burden not only on the affected individuals but also on society [1]. Tumor necrosis factor α (TNF-α) antagonists are used as monotherapy as well as in combination with conventional antirheumatic drugs such as metotrexate [2]. These antagonists block the interaction of TNF-α with its receptors on cell surface, thereby lowering the systemic and local levels of pro-inflammatory cytokines, preventing infiltration of leukocytes and lymphocytes to the sites of inflammation, promoting inhibition of nuclear factor-KB, inducing apoptosis of TNF-α-producing cells, lowering the level of endothelial adhesion molecules, and improving endothelial function. Still, about 30% of patients do not respond to treatment [3].

Patients with RA have an increased risk of atherosclerosis complications causing cardiovascular disease (CVD), increased atherosclerosis progress, and likely also plaque vulnerability, though there is a variation in reports [4, 5]. Biologics may prevent CVD in RA by ameliorating atherosclerosis complications [6].

Proprotein convertase subtilisin kexin 9 (PCSK9) was identified as a novel factor influencing LDL-metabolism. Genetic variants leading to low PCSK9 activity through missense variants and were associated with a low risk of CVD [7, 8] while other genetic variants led to high PCSK9 activity and high levels of LDL [9]. A mechanism was identified where PCSK9 targets the LDL-receptor (LDLR), and ensuing decreased activity of LDLR leads to increased LDL-levels and inhibition of PCSK9 is now used to treat CVD-patients [10–13].

Both statins and inhibition of PCSK9 may have other effects than LDL-lowering. They both inhibit oxidized LDL (OxLDL)-mediated immune activation through similar but not identical mechanisms [14, 15]. OxLDL, together with dead cells and activated immune competent cells, producing mainly proinflammatory cytokines are key elements of atherosclerotic plaques [16].

Earlier, we have reported that PCSK9-levels are raised among patients with high disease activity in systemic lupus erythematosus (SLE) and that OxLDL induced PCSK9 in dendritic cells (DC), effects which were significantly stronger in DCs from SLE patients than from controls [17]. OxLDL, which is increased in SLE, induced PCSK9, an effect which was higher among SLE patients. We suggested that PCSK9 could play an immunological role in SLE [17]. Also other studies demonstrate pro-inflammatory effects of PCSK9 [15, 18].

We here report that low PCSK9-levels at start of anti-TNF treatment in patients with RA are associated with being a responder to that therapy and elucidate potential underlying mechanisms. The implications are discussed.

## Materials and methods

### Patients and healthy individuals

One hundred sixty outpatients who satisfied the American College of Rheumatology (ACR) criteria for rheumatoid arthritis (RA) and were not previously treated with biologics were recruited for the study at the Rheumatology Department, Karolinska University Hospital Huddinge. The patients were administered anti-TNF-α (adalimumab, infliximab, or etanercept) for at least 1 year. The patients were followed and disease activity was assessed at treatment initiation time 0 and at 3, 6, and 12 months. Disease activity was assessed by the composite index disease activity score calculated in 28 joints (DAS28; range 0–9.4, best to worse). This composite index includes the number of the swollen joints, number of tender joints, and patient’s global assessment of disease activity measured on a visual analog and erythrocyte sedimentation rate (ESR). Remission was defined as DAS28 < 2.6 according to the European League Against Rheumatism (EULAR) criteria [19]. A Swedish version of the Stanford Health Assessment Questionnaire (HAQ) [20] was used to assess functional disability. In addition, information on body mass index (BMI; kg/m^2^), hypertension, rheumatoid factor (RF) positivity, current smoking, history of diabetes mellitus, CVD (myocardial infarction, congestive heart failure, angina pectoris, and ischemic stroke), and current medications was collected.

The ethics committee at the Karolinska Institute, Stockholm, Sweden, has approved this study. The study was performed according to the Helsinki declaration. All participants provided written informed consent.

### PCSK9 measures in serum

Sera were collected at baseline and preserved at − 80 °C until analysis was done. To separate serum, the blood was allowed to clot for 60 min at room temperature, then centrifuged 1000 g for 10 min.

Sera were diluted 100 times. Enzyme-linked immunosorbent assay (ELISA) was used to determine the PCSK9 level in all sera at baseline. ELISA was performed by a commercial kit (R&D Systems, UK) following the manufacturer’s protocol. PCSK9 levels were expressed as picogram per milliliter (pg/ml). The range of standard in ELISA kit was 125 pg–8000 pg/ml. So, after diluting 100 times, the range was within the standard.

### Cell culture

#### Macrophage culture

Buffy coats from healthy individuals were from the Karolinska University Hospital, Sweden. PBMCs were isolated from buffy coats, then CD14 positive monocytes were separated by Miltynei Biotech separation kit. The monocytes were cultured in RPMI media supplemented with 10%FBS and 40 ng/ml of GM-CSF. Differentiation was confirmed by microscopic observation and the expression of CD11b, where more than 98% cells of cells being differentiated. These differentiated macrophages were stimulated with various concentration of endotoxin-free PCSK9 (Sigma Aldrich) in the presence or absence of 5 μg/ml of anti-PCSK9 antibodies (Amgen) for 24 h. The experiments were performed at least three times with cells (macrophages) of three individuals (*n* = 3 in each experiment).

#### Synoviocytes

Human fibroblast-like synoviocytes from rheumatoid arthritis patients were purchased from Lonza and cell passage 1 or 2 were cultured with DMEM media with 10% FBS and stimulated with various concentration of PCSK9 in the presence or absence of anti-PCSK9 antibodies for 24 h. Anti-PCSK9 antibodies were provided by Amgen.

Experiments were performed three times and with triplicates in each experiment. Cytokines IL-1beta, TNF-α, and monocyte chemoattractant protein-1 (MCP-1) were measured from cell-cultured supernatants by ELISA duoset (R&D, Biotechne) according to manufacturer’s instruction. The experiments were performed with the cell passage numbers 1 and 2. All experiments were performed separately at least three times with at least triplicates in the separate experiments. Anti-PCSK9 antibodies were provided by Amgen.

### Statistical analysis

The association of PCSK9-levels with disease activity score of 28 joints (DAS28) and functional ability (HAQ scores) of the TNF–α antagonist-treated RA patients were assessed at baseline and after 3, 6, and 12 months. The data were analyzed by using generalized linear model. In order to obtain the association of PCSK9 at baseline with the outcomes of TNF–α antagonist treatment over time, we estimated the outcome (DAS28), at each follow-up.

Patients were categorized into quartiles (Q1, Q2, Q3, and Q4) based on the distribution of PCSK9 at baseline. For each follow-up (0 to 3, 0 to 6, and 0 to 12), variation in improvement of DAS28 within quartiles was calculated from crude models and models adjusted for potential confounding factors we chose to adjust for the same factors as in a larger previous study, sex, diabetes mellitus, hypertension, and CV history, which are known to be associated with PCSK9 [21]. Age was not associated with PCSK9 herein. The values of *p* < 0.05 were considered statistically significant. These analyses were performed using SAS 9.4 release (SAS Institute, Cary, NC).

Data from cell culture experiments were presented in a bar diagram with standard deviation. Statistical analysis was performed by one-way ANOVA.

## Results

### Patients’ demographic and clinical characteristics

Patients’ demographic and clinical characteristics are studied and summarized in Table 1. About three times higher number of patients (35.9%) in the first quartile (the lowest level of PCSK9) achieved remission after 1 year compared to those with the highest level of PCSK9 (12.8%) [risk ratio 0.36 (95% CI 0.15, 0.9)]. The odds of reaching remission in the lowest PCSK9 patients increased significantly (74%) compared to those at the highest PCSK9 category [odds ratio 0.26 (95% CI 0.08, 0.82). The characteristics of patients administered different anti-TNF-α drugs such as adalimumab, infliximab, and etanercept were similar (data not shown). There was no association between PCSK9 levels and DAS28 at baseline (data not shown).

**Table 1:** Clinical characteristics and baseline demographic of 160 RA patients

Age, yrs	56.2 ± 12.4
Female, *n* (%)	117 (72.2)
Duration of RA, yrs	7 (4–14)
RF-positivity, *n* (%)	132 (81.5)
Current smoking, *n* (%)	39 (24.1)
Hypertension, *n* (%)	90 (61.2)
Diabetes mellitus, *n* (%)	9 (5.6)
CVD comorbidity, *n* (%)	46 (28.4)
Statin use, *n* (%)	6 (3.7)
BMI, kg/m^2^	25.2 ± 4.6
DAS28	5.7 ± 1.0
HAQ-score	1.3 ± 0.6
MTX use, *n* (%)	126 (77.8)
GC use, *n* (%)	54 (33.3)
GC, mg/day	5 (5 to 7.5)
NSAID use, *n* (%)	112 (69.1)

Values are mean values with SD or median values with IQR as indicated

*RF* rheumatoid factor, *CVD* cardiovascular disease, *BMI* body mass index, *DAS28* disease activity score of 28 joints, *HAQ* health assessment questionnaire, *GC* glucocorticoid, *NSAID* non-steroidal anti-inflammatory drug, *yrs* years, *MTX* methotrexate

### Distribution of PCSK9 among quartiles in the RA patients before TNF–α antagonist treatment

Upon distribution of the patients into quartiles based on their baseline levels of PCSK9, the median values of PCSK9 in the first, second, third, and fourth quartile were determined to be 102.64 ng/ml (IQR 29.65), 141.29 ng/ml (IQR 22.12), 189.96 ng/ml (IQR 19.34), and 259.93 ng/ml (IQR 65.70), respectively.

### Association between baseline PCSK9 level and DAS28 in RA patients

**Fig. 1:**
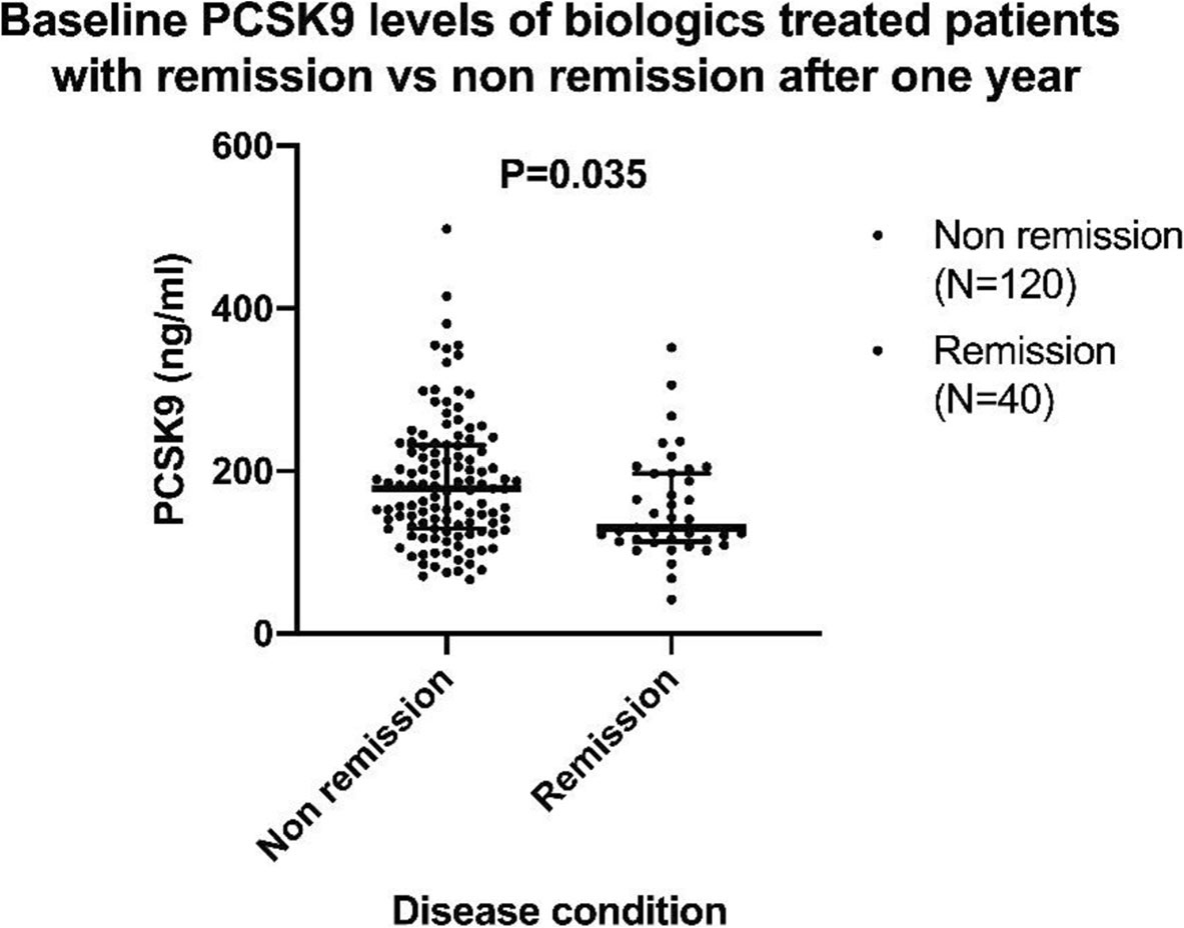
Results are presented as Scatter dot: median with interquartile range. PCSK9 levels at baseline among TNF–α antagonist-treated patients with remission (DAS < 2.6) vs non-remission (DAS ≥ 2.6) after 1 year. Results are presented as whiskers: min to max. Student’s *t* test was performed for statistical analysisk

After 1 year of treatment, DAS28 values were generally decreased, with 43 patients (27%) achieving remission target (DAS28 < 2.6). The PCSK9 levels of patients at baseline who achieved remission were compared with those not in remission after 1 year. A significantly lower level of PCSK9 level at baseline, *p* = 0.035, was observed in patients who reached remission after 1 year compared to those not in remission (Fig. 1).

In patients in the lowest PCSK9 category at baseline (first quartile, Q1), the mean DAS28 after 3 months was found to have 0.32, 0.46, and 0.45 units of greater reductions than the higher PCSK9 categories; second, third, and fourth quartiles (Q2, Q3, and Q4), respectively (Table 2). This was even greater after 6 months following the same clear trend of baseline PCSK9-dependent DAS28 change as it was after 3 months. At 12 months of TNF–α antagonist treatment, the mean DAS28 was found reduced in all PCSK9 categories but DAS reduction remained significantly greater in patients with the highest PCSK9 (Q4) compared to those at the lowest PCSK9 (Q1) in both crude (*p* = 0.01) and adjusted analysis (*p* = 0.004). Moreover, the mean DAS28 reduction was also found to be significantly higher in patients at Q3 compared to those at the lowest PCSK9 category in adjusted analysis (*p* = 0.03; Table 2).

**Table 2:** DAS28 values and number of patients who reached remission (DAS28 < 2.6) in the different PCSK9 quartiles

Period (month)	Q1 (*N* = 40)		Q2 (*N* = 40)		Q3 (*N* = 40)		Q4 (*N* = 40)	
	DAS28 (mean)	Remission (patients)	DAS28 (mean)	Remission (patients)	DAS28 (mean)	Remission (patients)	DAS28 (mean)	Remission (patients)
3	3.69	**8**	4.04	**3**	4.02	**3**	4.00	**5**
6	3.68	**8**	3.95	**5**	4.01	**5**	3.86	**3**
12	3.30	**14**	3.89	**9**	3.79	**10**	4.10	**5**

*DAS28* disease activity score of 28 joints, *Q1* first quartile, *Q2* second quartile, *Q3* third quartile, *Q4* fourth quartile, *N* number of patients

After 1 year 35.9% of patients at lowest PCSK9 category achieved remission, which was significantly higher compared to those at the highest PCSK9 category where only 12.8% achieved remission [risk ratio 0.36 95% CI 0.15, 0.9)] (Table 2).

A statistically significant increase in the odds of reaching remission in patients at the lowest PCSK9 category compared to those at the highest PCSK9 category was observed by logistic regression after 1-year treatment [odds ratio 0.26 (95% CI 0.08, 0.82 (Table 2).

### Associations between PCSK9 at baseline and ESR, CRP, and HAQ

Functional ability (HAQ) of patients in different categories also followed the same pattern except for those patients in Q3 category who experienced a greater mean reduction in both CRP, HAQ than the highest PCSK9 category but did not reach a statistical significance (Table 3).

**Table 3:** Crude and adjusted differences in mean reductions between quartiles at all follow up during TNF–α antagonist treatment for 1 year in RA patients

Model	Period (months)	Difference in mean decrease. Q1–Q2	*P* value	Difference in mean decrease. Q1–Q3	*P* value	Difference in mean decrease. Q1–Q4	*P* value
**DAS28**							
**Crude**	0 to 3	0.3	0.27	0.43	0.13	0.37	0.18
	0 to 6	0.29	0.34	0.49	0.1	0.31	0.32
	0 to 12	0.56	0.1	0.67	0.06	0.91	**0.01**
**Adjusted**	0 to 3	0.32	0.23	0.46	0.11	0.45	0.12
	0 to 6	0.37	0.22	0.53	0.08	0.48	0.13
	0 to 12	0.65	0.06	0.76	**0.03**	1.06	**0.004**
**CRP**							
**Crude**	0 to 3	6.63	0.39	6.73	0.39	13.61	0.08
	0 to 6	3.11	0.63	2.82	0.67	5.66	0.39
	0 to 12	7.67	0.39	10.68	0.23	9.26	0.31
**Adjusted**	0 to 3	6.07	0.44	5.16	0.52	13.79	0.09
	0 to 6	3.67	0.58	3.04	0.65	6.98	0.31
	0 to 12	8.56	0.32	11.64	0.19	10.32	0.25
**ESR**							
**Crude**	0 to 3	0.14	0.96	1.9	0.6	5.89	0.11
	0 to 6	−1.87	0.64	1.07	0.79	0.68	0.86
	0 to 12	0.95	0.84	5.81	0.22	7.28	0.13
**Adjusted**	0 to 3	0.17	0.96	2.1	0.58	5.39	0.15
	0 to 6	−1.77	0.66	0.78	0.85	1.01	0.81
	0 to 12	1.43	0.76	6.87	0.16	6.98	0.15
**HAQ**							
**Crude**	0 to 3	−0.03	0.98	2.84	0.16	−0.06	0.97
	0 to 6	0.03	0.76	0.08	0.4	−0.003	0.97
	0 to 12	0.07	0.51	0.99	0.39	0.08	0.47
**Adjusted**	0 to 3	−0.03	0.98	2.81	0.18	0.05	0.98
	0 to 6	0.06	0.55	0.12	0.26	0.03	0.75
	0 to 12	0.1	0.36	0.13	0.24	0.14	0.25

Results are indicated as mean and the number of patients is as indicated in Table 1. *P* values are calculated from the difference in disease outcomes at different quartiles compared to Q1. Adjustment was done for sex, diabetes mellitus, hypertension, and CV history

*DAS28* disease activity score of 28 joints, *HAQ* health assessment questionnaire, *CRP* C-reactive protein, *ESR* erythrocyte sedimentation rate, *Q1* first quartile, *Q2* second quartile, *Q3* third quartile, *Q4* fourth quartile

### PCSK9 induced pro-inflammatory effects and inhibition by anti-PCSK9 antibodies

**Fig. 2:**
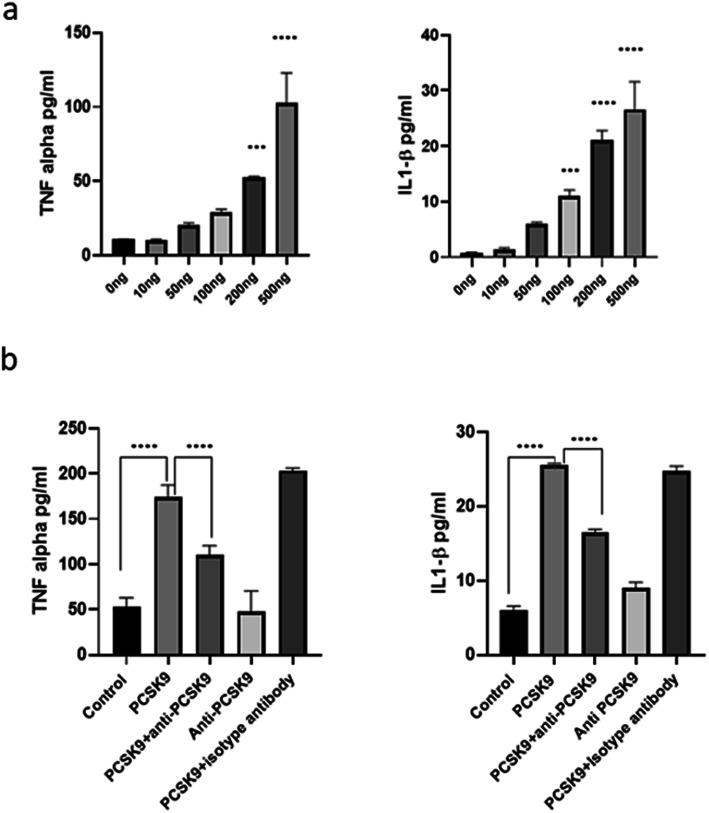
Macrophages were stimulated with various concentration of PCSK9 in the presence or absence of anti-PCSK9 antibodies for 24 h. **a** Macrophages induced TNF-alpha and IL-1beta (*n* = 3) in a concentration-dependent manner. **b** Macrophages were stimulated with 500 ng/ml of PCSK9 in the presence or absence of 5 μg/ml of anti-PCSK9 antibodies for 24 h. The level of TNF-alpha and IL-1beta was suppressed by anti-PCSK9 antibodies (*n* = 3). *P* value ≤ 0.0005 was considered *** and ≤ 0.0001 was condsidered as ****

PCSK9 induced TNF-α and IL-1beta in macrophages in a dose-dependent way (Fig. 2a), and this was inhibited by anti-PCSK9 antibodies (Fig. 2b). The experiments were performed three times with cells obtained from three different individual donors, and experiments on cells of all donors showed similar results. Experiments from cells of one donor are showed as a representative, and experiments with cells from other donors are presented in the supplementary figure 1 and 2.

**Fig. 3:**
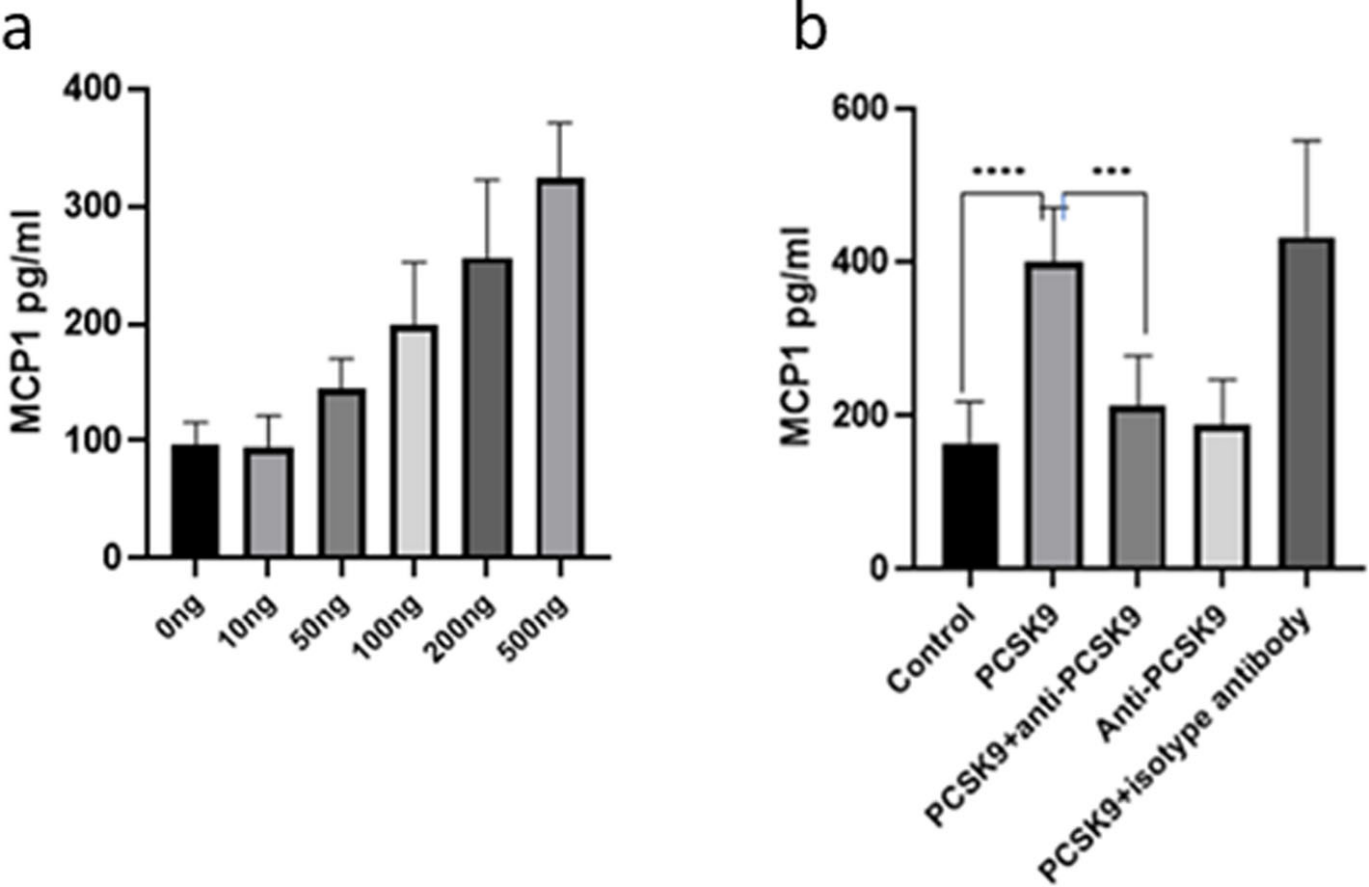
Synoviocytes were cultured with or without various concentration of PCSK9 in the presence or absence of anti-PCSK9 antibodies for 24 h. Experiments were performed three times and triplicate in each time. The mean value of 3 independent experiments was presented in the bar diagram. **a** PCSK9 induced level of MCP1 in the synoviocytes in a concentration-dependent manner (*n* = 9). **b** Synoviocytes were stimulated with 500 ng/ml of PCSK9 in the presence or absence of 5 μg/ml of anti-PCSK9 antibodies for 24 h, and the level of MCP1 was inhibited by anti-PCSK9 antibodies (*n* = 9). *P* value ≤ 0.0005 was considered *** and ≤ 0.0001 was condsidered as ****

PCSK9 induced MCP1 in synoviocytes in a dose-dependent way (Fig. 3a), and this effect was inhibited by anti-PCSK9 antibodies (Fig. 3b). The experiments on synoviocytes (passages 1 and 2) were performed three times, and the mean value of three experiments is presented. TNF-α could not be detected from synoviocytes.

## Discussion

We here report that baseline levels of PCSK9 were negatively associated with disease activity as determined by DAS28 after 3 months, 6 months, and 12 months among TNF–α antagonist-treated RA patients. The odds ratio after 1 year to be in remission among RA patients in the lowest quartile of PCSK9-levels at baseline was four times higher than for those in the highest quartile of PCSK9 levels.

Despite advances during recent years, the treatment of RA remains a challenge. Anti-TNF-α therapy is the most common biologic treatment for RA patients, but the rate of remission is not satisfactory and about 30% of patients are non-responders [22].

Our findings could have clinical implications, both to improve prediction of effects of TNF-α-inhibition in RA and to improve prediction of outcome in order to optimize therapy, because one of the problems is the difficulty to identify non-responders before treatment. Our findings may imply that RA patients with low baseline levels of circulating PCSK9 could respond to TNF–α antagonist treatment significantly better than those with higher baseline PCSK9.

It is also possible that RA-patients with high PCSK9 levels could be eligible for other types of therapy, or combinations with TNF-α-inhibition at an early stage, depending on whether other treatments, as established disease modifying anti-rheumatic drugs (DMARDs) or novel biological therapies are associated with baseline PCSK9-levels.

Another question is whether PCSK9 and its inhibition also could play a role in underlying mechanisms and in immune reactions and inflammation causing RA. We therefore investigated the effects of PCSK9 on activation of cell types which are implicated in RA-synovia and generally believed to play an important role in disease development.

In the synovial joint of RA patients, activated macrophages, neutrophils, and lymphocytes are abundant and play an important role in the disease pathogenesis and most likely also activated synoviocytes play an essential role in local inflammation [23, 24]. To investigate potential underlying mechanism of anti-TNF-α treatment failure, we studied effects of PCSK9, in physiological concentrations in similar range as those present in the circulation, on macrophages and synoviocytes.

We determined that PCSK9 induces pro-inflammatory TNF-α and IL-1-beta in a concentration-dependent manner from macrophages. The finding could thus shed light on the lack of response to anti-TNF-α therapy in RA patients with high PCSK9 levels, since PCSK9 could counter the anti-TNF-α effects. We could not detect TNF-α from the synoviocytes.

MCP1 may play an important role in RA pathogenesis, including recruitment of macrophages [25]. Further, inhibition of MCP-1 ameliorated arthritis in rat models [26]. Also levels of MCP-1 in sera of RA-patients are raised [25]. Still the role and induction of MCP-1 in RA is poorly understood. We here report that PCSK9 induced MCP-1 from synoviocytes, and it is thus possible that PCSK9 could contribute to MCP-1 induction at least in subgroups of RA patients. We speculate that PCSK9-induced synoviocytes recruit leucocytes including macrophages through MCP-1 to the site of inflammation and thus increasing the risk of failed anti-TNF-α therapy.

These effects—induction of TNF-α and IL-1 beta from macrophages and MCP-1 in synoviocytes, were inhibited by antibodies against PCSK9. It is thus possible that inhibition of PCSK9 could contribute to amelioration of chronic inflammation as in RA, especially in individuals with high levels of PCSK9. Clinical studies are needed to study this possibility. These findings could also shed light on the increased risk of atherosclerosis and CVD which is described in RA, and also on the inflammation in atherosclerotic plaques.

A combination of traditional risk factors, as diabetes, hypertension, and smoking and non-traditional risk factor as inflammation may account for the increased risk of CVD including stroke and myocardial infarction (MI).

Atherosclerosis is characterized by the presence of activated immune competent cells, but also necrotic core of dead cells and OxLDL, mostly engulfed in foam cells [16]. OxLDL induces activation of monocytes and T cells [27, 28], also from human atherosclerotic plaque-derived T cells and dendritic cells (DC) [29]. OxLDL has been detected in foam cells in rheumatic synovia [30], and OxLDL in the circulation is raised in RA and also associated with CVD in RA [31].

In previous studies, we demonstrated that OxLDL-induced pro-inflammatory cytokines IFN-δ and IL-17 in a complex immune reaction where heat shock protein 60/65 plays a role [30]. PCSK9 plays a key role in OxLDL-induced pro-inflammatory effect by dendritic cells (DCs) [15]. PCSK9-silencing inhibited OxLDL-induced activation of DCs and subsequently induction of regulatory T cells (T-regs) and IL-10 cytokines [15].

T-reg cells could be of major importance in autoimmune diseases, such as RA, by suppressing several immune cells, including CD8+ and CD4+ T-cells, antigen presenting cells (APCs), natural killer (NK) cells, and dendritic cells [32]. Therefore, RA ameliorating T-reg cells and anti-inflammatory cytokine IL-10 demolishing effects of PCSK9 could contribute to modification of anti TNF–α treatments in RA patients.

There are limitations to this study. We were not able to differentiate between three different TNF-α-antagonists due to the size of the study population, and it would be interesting to investigate also other novel treatment modalities in RA. Further, the numbers of patients in remission per studied PCSK9 quartile are relatively low. The in vitro experiments are hypothesis-generating, but studies with PCSK9 inhibition in RA are necessary to establish PCSK9 as an underlying agent in this disease (and other inflammatory conditions). We only followed-up patients for 1 year. A longer follow-up time is needed to establish if the associations remain between disease activity and outcome, as here in DAS28, but also for other parameters related to the disease including HAQ.

## Conclusions

Taken together, the results indicate that PCSK9 could play a role in predicting outcome in RA and also that PCSK9 inhibition could be of interest therapeutically in RA, a possibility deserving further studies.

## Supplementary Information

**Additional file 1: Supp. Fig-1.** Repeated experiments of Fig. 2a using cells from different donors. **Supplemental Figure-1.** Similar results were observed in experiments using cells from different donors. TNF-alpha and IL-1beta induced in response to various concentration of PCSK9. *P* value ≤0.005 was considered **, ≤ 0.0005 was considered *** and ≤ 0.0001 was condsidered as ****.

**Additional file 2:** **Supp. Fig- 2.** Repeated experiments of figure 2b using cells from different donors. **Supplemental Figure-2.** Similar results were obtained in experiments using cells from different donors. PCSK9-indued TNF-alpha and IL-1beta in macrophages were suppressed by anti-PCSK9 antibodies. P value ≤ 0.05 was considered * and ≤ 0.005 was considered as **, and ≤ 0.0001 was considered ****.

## Data Availability

The datasets used and/or analyzed during the current study are available from the corresponding author on reasonable request.

## Abbreviations

PCSK9: Proprotein convertase subtilisin kexin 9
LDL: Low-density lipoprotein
Ox: Oxidized
TNF: Tumor necrosis factor
RA: Rheumatoid arthritis
CRP: C-reactive protein
MCP-1: Monocyte chemoattractant protein-1
DAS 28: Disease activity score 28
HAQ: Health Assessment Questionnaire
