# Supplementary material for: Low levels of PCSK9 are associated with remission in patients with rheumatoid arthritis treated with anti-TNF-α: potential underlying mechanisms
Source: Arthritis Res Ther. 2021 Jan 19;23:32. doi: 10.1186/s13075-020-02386-7 (PMC7814540; doi:10.1186/s13075-020-02386-7)
Supplement: Supplementary file 1 — Additional file 1: Supp. Fig-1. Repeated experiments of Fig. 2a using cells from different donors. Supplemental Figure-1. Similar results were observed in experiments using cells from different donors. TNF-alpha and IL-1beta induced in response to various concentration of PCSK9. P value ≤0.005 was considered **, ≤ 0.0005 was considered *** and ≤ 0.0001 was condsidered as ****. [file 13075_2020_2386_MOESM1_ESM.docx]

Supp. Fig-1: Repeated experiments of figure 2-A using cells from different donors.


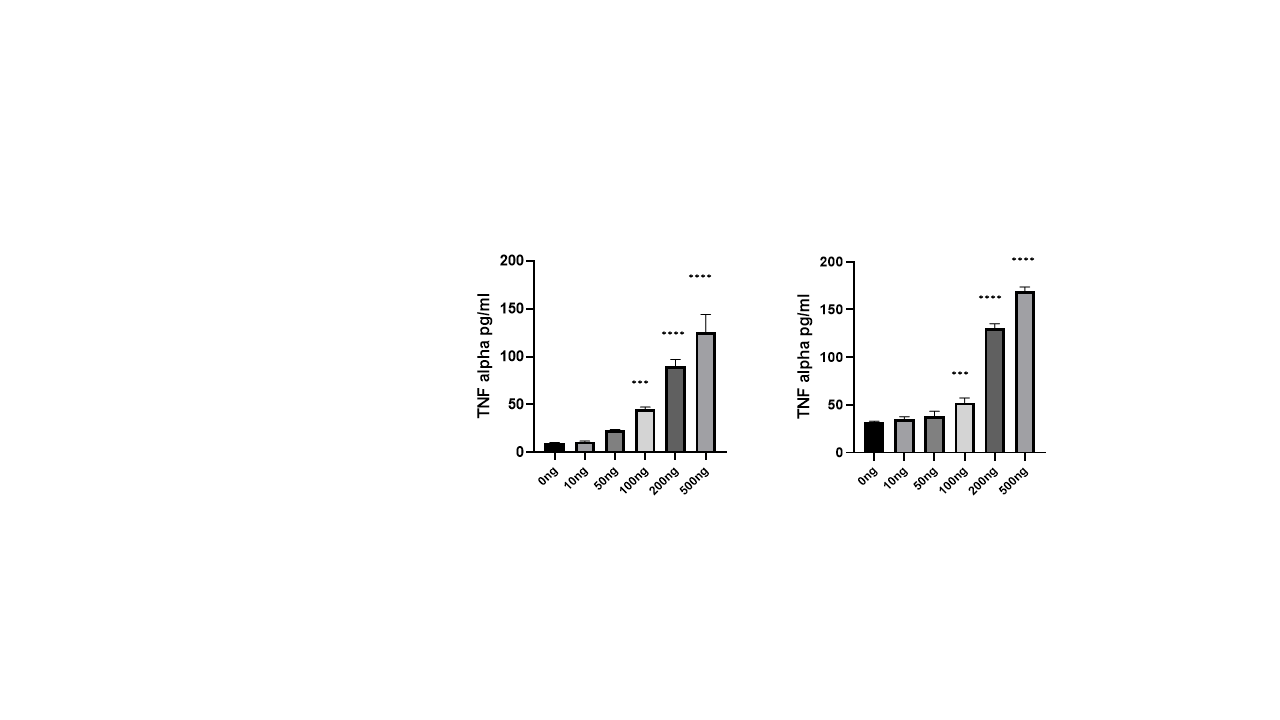


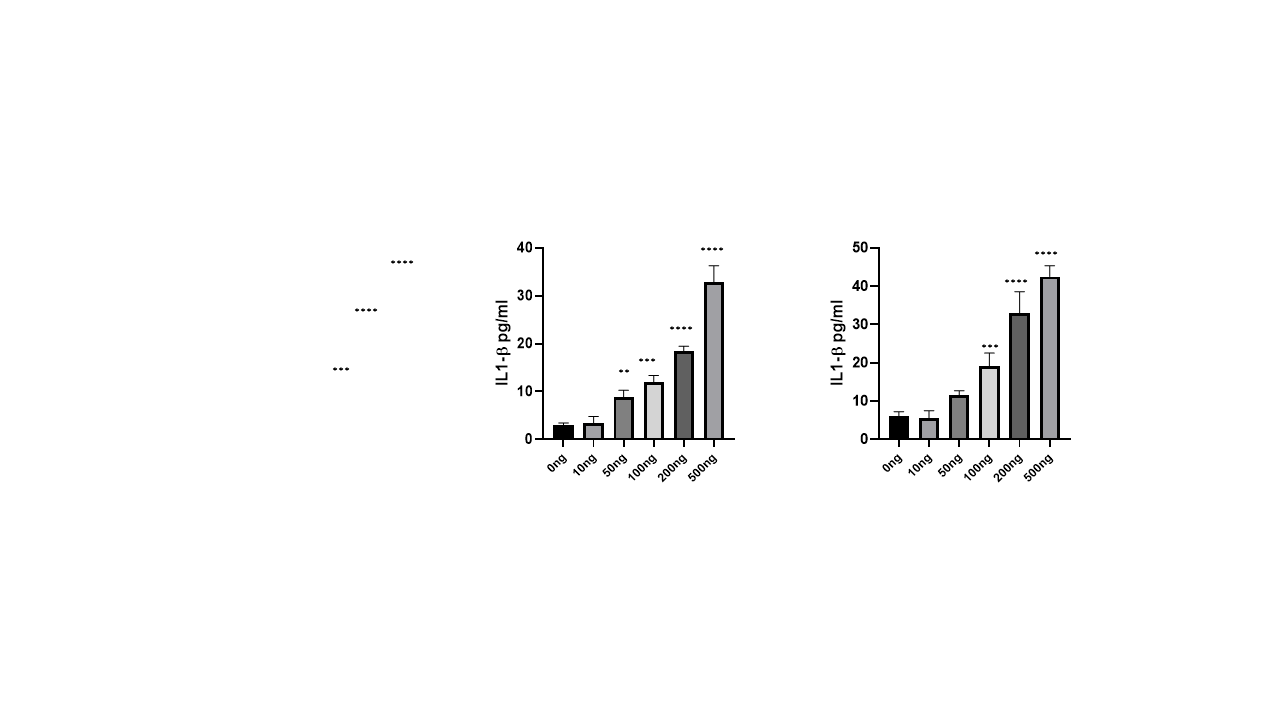


Supplemental Figure-1: Similar results were observed in experiments using cells from different donors. TNF-alpha and IL-1beta induced in response to various concentration of PCSK9. P value ≤ 0.005 was considered **, ≤ 0.0005 was considered *** and ≤ 0.0001 was condsidered as ****.
