# Supplementary material for: Low levels of PCSK9 are associated with remission in patients with rheumatoid arthritis treated with anti-TNF-α: potential underlying mechanisms
Source: Arthritis Res Ther. 2021 Jan 19;23:32. doi: 10.1186/s13075-020-02386-7 (PMC7814540; doi:10.1186/s13075-020-02386-7)
Supplement: Supplementary file 2 — Additional file 2: Supp. Fig- 2. Repeated experiments of figure 2b using cells from different donors. Supplemental Figure-2. Similar results were obtained in experiments using cells from different donors. PCSK9-indued TNF-alpha and IL-1beta in macrophages were suppressed by anti-PCSK9 antibodies. P value ≤ 0.05 was considered * and ≤ 0.005 was considered as **, and ≤ 0.0001 was considered ****. [file 13075_2020_2386_MOESM2_ESM.docx]

Supp. Fig- 2: Repeated experiments of figure 2-B using cells from different donors.


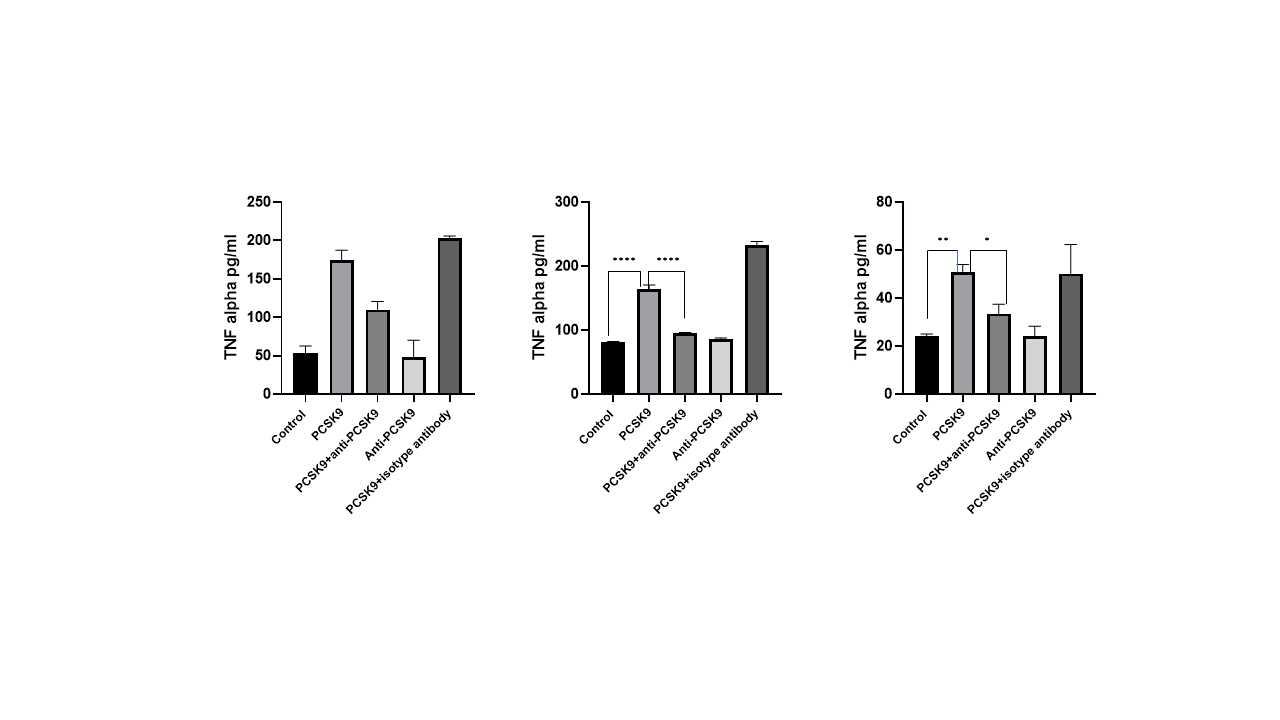


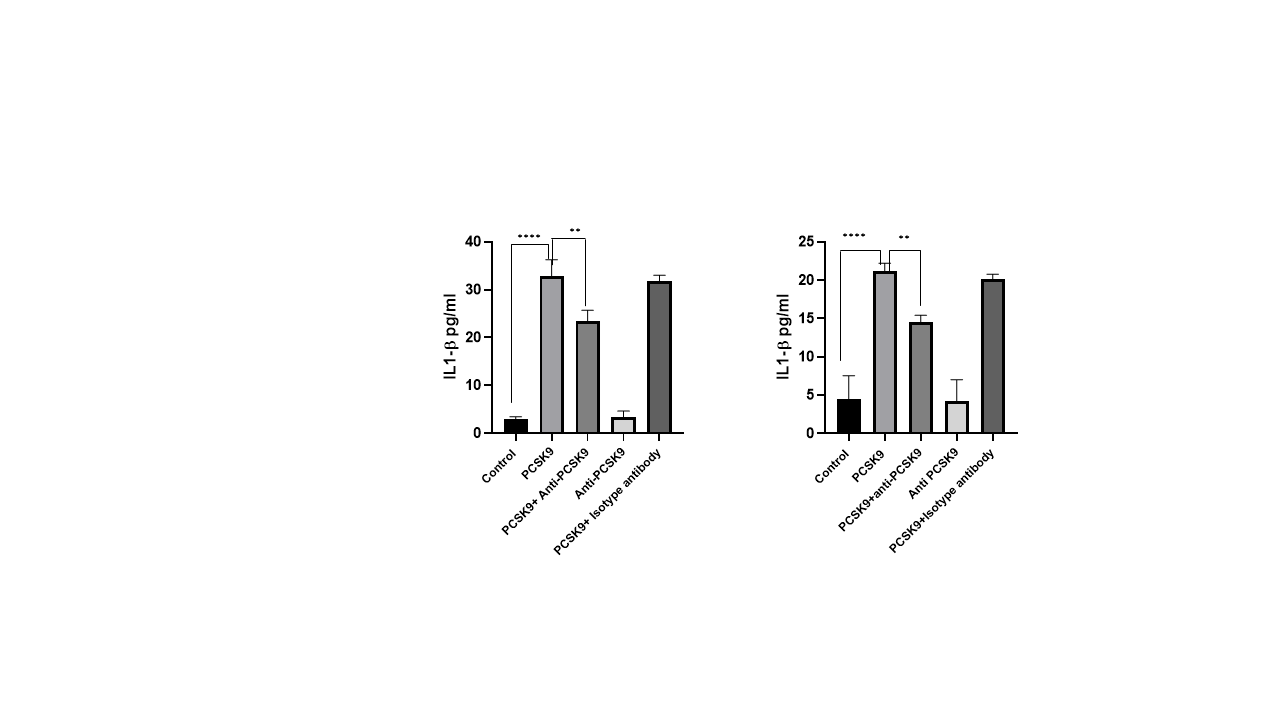


Supplemental Figure-2: Similar results were obtained in experiments using cells from different donors. PCSK9-indued TNF-alpha and IL-1beta in macrophages were suppressed by anti-PCSK9 antibodies. P value ≤ 0.05 was considered * and ≤ 0.005 was considered as **, and ≤ 0.0001 was considered ****.
